# Remnant cholesterol for diabetic kidney disease risk stratification in type 2 diabetes: a machine learning-based prevention tool

**DOI:** 10.3389/fnut.2025.1697943

**Published:** 2025-11-11

**Authors:** Yuehong Dai, Qi Pan, Yujie Yu, Yongjun Ma, Guangming Chen, Huabin Wang

**Affiliations:** 1 Department of Clinical Laboratory, Affiliated Jinhua Hospital, Zhejiang University School of Medicine, Jinhua, Zhejiang, China; 2 Nottingham Ningbo China Beacons of Excellence Research and Innovation Institute, University of Nottingham, Ningbo, China; 3 School of Laboratory Medicine and Life Science, Wenzhou Medical University, Wenzhou, Zhejiang, China; 4 Department of General Practice, Affiliated Jinhua Hospital, Zhejiang University School of Medicine, Jinhua, Zhejiang, China

**Keywords:** remnant cholesterol, diabetic kidney disease, type 2 diabetes, random survival forest, risk prediction model

## Abstract

**Background:**

Disturbances in lipid metabolism play a critical role in the onset and progression of diabetic kidney disease (DKD). Remnant cholesterol (RC), a marker of remnant lipoprotein metabolism, is an established cardiovascular residual risk factor. However, evidence linking RC to the risk of incident DKD is limited. This study aimed to investigate the association between RC and incident DKD and to develop a risk prediction model incorporating RC and other clinical variables in patients with type 2 diabetes (T2D).

**Methods:**

A retrospective cohort study of 2,122 patients with T2D and without baseline DKD was conducted. The association between RC and DKD risk was examined using multivariable Cox regression and restricted cubic spline (RCS) analysis. A random survival forest (RSF) algorithm was applied to identify potential predictors, followed by multicollinearity assessment. A RSF-based prediction model was developed and evaluated for discrimination, calibration, and clinical utility.

**Results:**

During a median follow-up of 4.22 years, 435 participants (20.5%) developed DKD. Higher RC quartiles were associated with an increased risk of DKD across all models; however, the hazard ratios for Q2 to Q4 were numerically similar, indicating the absence of a clear linear dose–response pattern. RCS analysis revealed a nonlinear association between RC and DKD risk (P for nonlinearity = 0.031), characterized by a steep initial increase followed by a plateau at higher RC levels. RSF identified 14 predictors (including ACR, RC) with no significant multicollinearity (all the variance inflation factors < 3). The model exhibited strong discrimination (3-year AUC = 0.86, 5-year AUC = 0.91) and calibration (3-year mean absolute error = 0.011, 5-year mean absolute error = 0.026), and outperformed “treat-all”/“treat-none” strategies in decision curve analysis.

**Conclusion:**

RC was independently and nonlinearly associated with DKD risk in T2D. The RSF model demonstrated good predictive performance and may assist individualized risk assessment and management.

## Introduction

Diabetic kidney disease (DKD) is one of the most common and devastating microvascular complications of type 2 diabetes (T2D), affecting up to 30–40% of patients during their lifetime and it represents a leading cause of end-stage renal disease worldwide (1–3). The development of DKD not only markedly increases the risk of cardiovascular morbidity and mortality but also imposes a substantial economic and social burden on healthcare systems (4, 5). Given that DKD is often clinically silent in its early stages, by the time microalbuminuria or a decline in estimated glomerular filtration rate (eGFR) become detectable, irreversible renal damage may have already occurred (1, 6). Therefore, early identification of individuals at high risk for DKD and timely intervention are essential to slowing disease progression, preventing kidney failure, and improving long-term outcomes in T2D populations (7–9).

Accumulating evidence suggests that disturbances in lipid metabolism play a critical role in the onset and progression of DKD (10–12). Abnormal lipid profiles promote renal injury through lipid deposition in glomerular and tubular cells, induction of oxidative stress, and activation of pro-inflammatory pathways, thereby accelerating glomerulosclerosis and tubulointerstitial fibrosis (10, 13). Notably, remnant cholesterol (RC)—calculated as total cholesterol minus high-density lipoprotein cholesterol (HDL-C) and low-density lipoprotein cholesterol (LDL-C)—primarily reflects atherogenic remnant lipoproteins, including very-low-density lipoprotein cholesterol (VLDL-C) and intermediate-density lipoprotein cholesterol (IDL-C) (14, 15). It serves as a marker of remnant lipoprotein metabolism, potentially capturing atherogenic lipid burden not reflected by conventional lipid parameters (14, 15). RC is increasingly recognized as a contributor to cardiovascular residual risk independent of LDL-C, contributing to atherosclerosis through endothelial dysfunction and plaque instability (15–17). Additionally, Wang et al. have confirmed that elevated RC is independently associated with increased arterial stiffness (18). As an early marker of systemic vascular damage, arterial stiffness suggests that RC may impair both macrovascular and microcirculatory systems through endothelial dysfunction and decreased vascular compliance (19). In the kidneys, RC-driven vascular stiffening may accelerate injury via dual pathways: by promoting glomerular capillary hypertension transmission and oxidative stress on one hand (20), and by activating fibrotic pathways through direct lipotoxic deposition in renal tissues on the other (21). However, clinical evidence linking RC to DKD remains relatively limited.

Therefore, a retrospective cohort study was designed in T2D patients without DKD at baseline to examine the association between RC and the risk of developing DKD, including an assessment of potential dose–response relationships. In addition, a DKD risk prediction model was developed and validated using machine learning approaches that incorporate RC with other routinely available clinical variables, aiming to enhance individualized risk stratification and inform targeted prevention strategies.

## Materials and methods

### Study population

**Figure 1:**
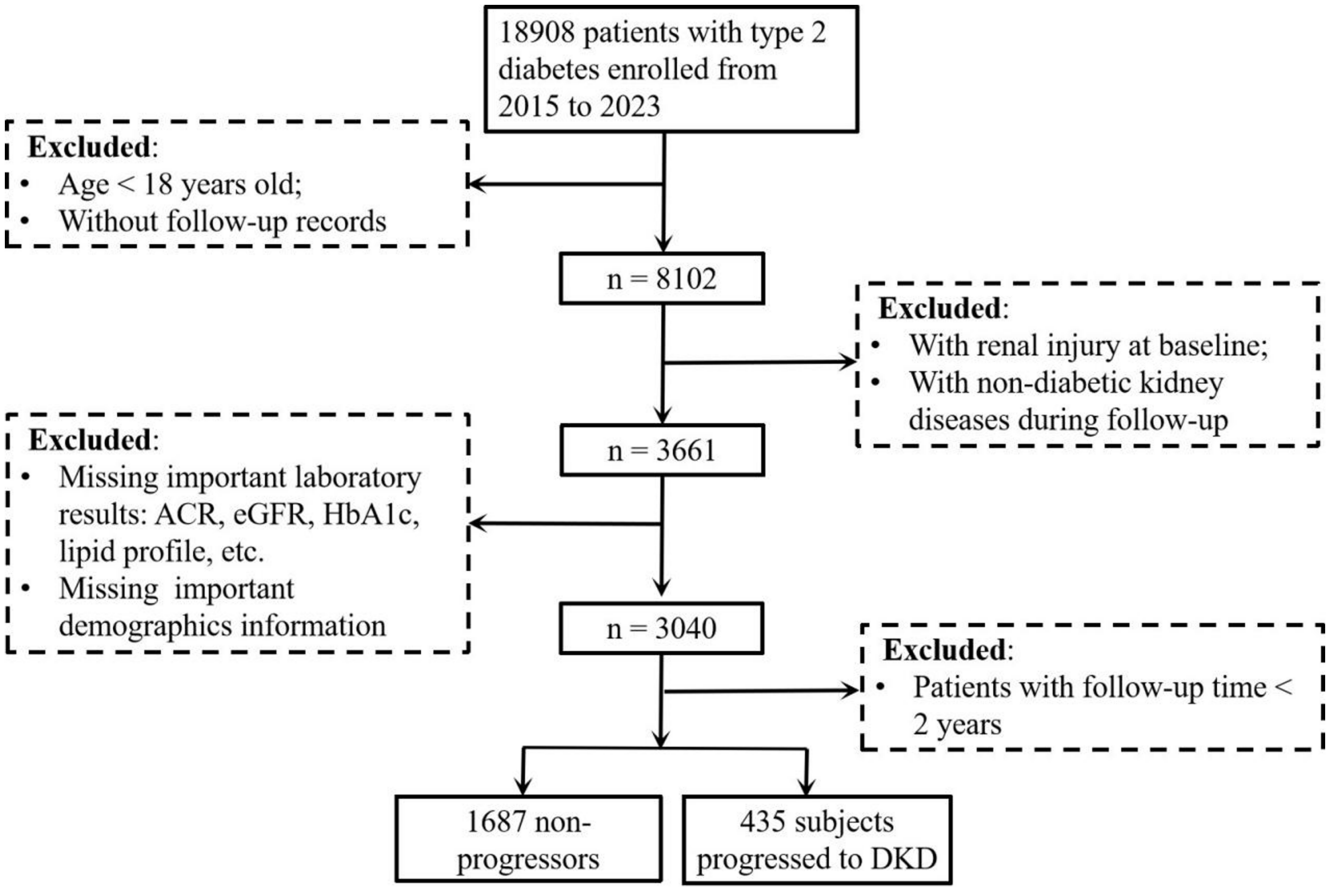
Study flow diagram of participant enrollment and exclusion for DKD risk analysis in patients with type 2 diabetes. The flow diagram showed the enrollment of patients with type 2 diabetes (2015–2023), exclusion criteria (age < 18 years, missing follow-up records, baseline renal injury, non-diabetic kidney disease during follow-up, follow-up time < 2 years, missing key laboratory/demographic data), and final cohort (1,687 non-progressors vs. 435 progressors to DKD) for analyzing DKD risk. DKD, diabetic kidney disease; ACR, albumin-to-creatinine ratio; eGFR, estimated glomerular filtration rate; HbA1c, glycated hemoglobin.

This retrospective cohort study included patients with T2D who were treated at the Affiliated Jinhua Hospital, Zhejiang University School of Medicine between January 2015 and December 2023. Eligible participants were identified through the hospital’s electronic medical record system. The inclusion criteria were: (1) age ≥18 years; (2) confirmed diagnosis of T2D; and (3) available follow-up data. Patients were excluded if they: (1) lacked follow-up records; (2) were missing key laboratory data [including urinary albumin-to-creatinine ratio (ACR), eGFR, glycated hemoglobin (HbA1c), and lipid profile] or important demographic information; (3) had renal injury (ACR ≥ 30 mg/g and/or eGFR <60 mL/min/1.73 m^2^) at baseline or developed non-diabetic kidney diseases during follow-up; or (4) had a follow-up duration of less than 2 years. A total of 2,122 patients with T2D were included in the final analysis (Figure 1). This study followed the tenets of the Declaration of Helsinki and was approved by the Ethics Committee of the Affiliated Jinhua Hospital, Zhejiang University School of Medicine (Approval No. [Res] 2025-Ethical Review-226). The requirement for informed consent was waived due to the retrospective nature of the study.

### Data collection

Demographic characteristics, medical history, medication use, and laboratory parameters were extracted from the electronic medical record system. The collected variables included: age, sex, smoking status, alcohol consumption, duration of diabetes, systolic blood pressure (SBP), diastolic blood pressure (DBP), body mass index (BMI), total cholesterol (TC), triglycerides (TG), LDL-C, HDL-C, fasting plasma glucose (Glu), uric acid (UA), glycated hemoglobin (HbA1c), serum creatinine (sCr), total protein (TP), serum albumin. Comorbidities and medication history, including hypertension status, use of angiotensin-converting enzyme inhibitors (ACEIs) or angiotensin II receptor blockers (ARBs), insulin, fibrates/statins, and sodium-glucose cotransporter 2 inhibitors/glucagon-like peptide-1 receptor agonists (SGLT2i/GLP-1RA), were also recorded. Then, the values of eGFR, ACR, and RC were calculated. eGFR was calculated based on sCr using the Xiangya equation, which is validated in Chinese populations (22). ACR was calculated by dividing urinary albumin concentration by urinary creatinine concentration (3). RC was calculated using the formula: RC = TC - HDL-C - LDL-C (14).

### Definitions

T2D was diagnosed according to World Health Organization guidelines (fasting plasma glucose ≥7.0 mmol/L or HbA1c ≥ 6.5%) (23). Hypertension was defined as systolic blood pressure ≥140 mmHg and/or diastolic blood pressure ≥90 mmHg, or current use of antihypertensive medications. Smoking and alcohol use status were categorized based on self-reported data from admission questionnaires. According to American Diabetes Association diagnostic criteria, DKD was defined as the development of eGFR <60 mL/min/1.73 m^2^ and/or ACR ≥ 30 mg/g (24, 25).

### Statistical analysis

All statistical analyses were conducted using R software (version 4.3.2), with two-sided *p* values < 0.05 considered statistically significant. Baseline characteristics were summarized as mean ± standard deviation for normally distributed continuous variables, median (interquartile range) for skewed data, and frequency (percentage) for categorical variables. Participants were stratified into quartiles of RC levels (Q1–Q4) with the following thresholds and group sizes: Q1: <0.46 mmol/L (*n* = 508), Q2: 0.47–0.63 mmol/L (*n* = 537), Q3: 0.64–0.82 mmol/L (*n* = 541), Q4: >0.83 mmol/L (*n* = 536). *p* values for trend across quartiles were tested using linear regression for continuous variables and the Cochran–Armitage test for categorical variables. Cox proportional hazards models were selected to evaluate RC-DKD associations. The covariate selection was based on major demographic characteristics (including age, sex, and BMI) and established DKD risk factors reported in previous studies (26–28). We constructed three models with adjustments for major covariables: Model 1 was adjusted for age and sex; Model 2 included Model 1 variables plus SBP, BMI, ACR, sCr, and HbA1c; and Model 3 further included diabetic duration, hypertension status, fibrate/statin use, and SGLT2i/GLP-1RA use. In the variable screening process for predictive modeling, TC, HDL-C, and LDL-C were not included because RC, which was selected for analysis, was calculated from these parameters, and their simultaneous inclusion could introduce multicollinearity. Similarly, given that nearly all participants receiving ACEIs/ARBs had hypertension in this study, hypertension status was included in the screening model, whereas ACEI/ARB use was not, in order to avoid redundancy and collinearity. Restricted cubic spline (RCS) models, adjusted for covariates in Model 3, were used to explore potential nonlinear associations between RC and DKD risk. For predictive modeling, the random survival forest (RSF) algorithm was applied, given its ability to handle right-censored survival data, capture nonlinear effects, and model complex interactions among predictors. The RSF was implemented via the randomForestSRC package, using 1,000 trees and optimizing terminal node size by minimizing the out-of-bag (OOB) error rate. Predictor importance was quantified using permutation-based mean decrease in the concordance index. Multicollinearity among RSF-selected variables was assessed using variance inflation factors (VIF) and Spearman correlation heatmaps. Model performance was evaluated by time-dependent receiver operating characteristic (ROC) curves, calibration plots generated from 1,000 bootstrap resamples, and decision curve analysis (DCA) to quantify the net clinical benefit compared with treat-all and treat-none strategies.

## Results

### Baseline characteristics of the study population

The baseline clinical and demographic characteristics of the 2,122 participants without DKD at baseline, stratified by quartiles of RC, were summarized in Table 1. The mean age of the overall study population was 57.47 ± 11.19 years, and 1,325 (62.4%) participants were male. During a median follow-up of 4.22 years, 435 participants (20.5%) developed incident DKD. Participants in higher RC quartiles were generally younger, had shorter diabetes duration, and higher values of BMI, SBP, DBP, TG, TC, LDL-C, fasting Glu, HbA1c, serum UA, and eGFR, along with lower HDL-C levels (all P for trend < 0.001). In addition, the prevalence of hypertension and the use of ACEI/ARB, insulin or fibrate/statin therapy decreased significantly across RC quartiles (all P for trend *p* < 0.05). However, a significant upward trend was also observed for smoking status. No significant differences were observed in sex distribution, drinking status, sCr, TP, albumin, ACR, or use of SGLT2i/GLP-1RA (all P for trend > 0.05).

**Table 1:** Baseline characteristics of study population according to RC quartiles.

Variable	Total (*n* = 2,122)	Q1 (*n* = 508)	Q2 (*n* = 537)	Q3 (*n* = 541)	Q4 (*n* = 536)	*P* for trend
Age, year	57.47 ± 11.19	60.31 ± 10.56	59.05 ± 10.37	56.56 ± 10.10	54.10 ± 11.73	<0.001
Gender, *n* (%)						
Female, *n* (%)	797 (37.56)	190 (37.40)	210 (39.11)	212 (39.19)	185 (34.52)	0.352
Male, *n* (%)	1,325 (62.44)	318 (62.60)	327 (60.89)	329 (60.81)	351 (65.48)	
Smoking, *n* (%)	788 (37.14)	175 (34.45)	192 (35.75)	201 (37.15)	220 (41.05)	0.024
Drinking, *n* (%)	775 (36.52)	189 (37.21)	191 (35.57)	194 (35.86)	201 (37.50)	0.889
Diabetic duration, year	7.00 (3.00, 10.00)	10.00 (4.00, 15.00)	7.00 (4.00, 10.00)	6.00 (3.00, 10.00)	5.00 (1.00, 10.00)	<0.001
SBP, mmHg	133.91 ± 18.05	132.35 ± 18.56	133.95 ± 18.28	134.43 ± 17.12	134.80 ± 18.14	0.028
DBP, mmHg	78.87 ± 11.24	76.23 ± 10.54	78.32 ± 11.24	79.95 ± 11.11	80.86 ± 11.46	<0.001
BMI, kg/m^2^	24.65 ± 4.35	23.72 ± 3.49	24.48 ± 4.26	24.85 ± 3.96	25.51 ± 5.26	<0.001
TC, mmol/l	4.70 ± 1.12	4.03 ± 0.86	4.28 ± 0.86	4.78 ± 0.84	5.65 ± 1.14	<0.001
TG, mmol/l	1.47 (1.03, 2.20)	1.00 (0.78, 1.29)	1.29 (0.98, 1.72)	1.64 (1.23, 2.24)	2.48 (1.71, 3.76)	<0.001
LDL, mmol/l	2.88 ± 0.82	2.46 ± 0.75	2.62 ± 0.72	2.98 ± 0.67	3.44 ± 0.78	<0.001
HDL, mmol/l	1.12 ± 0.30	1.23 ± 0.31	1.12 ± 0.27	1.08 ± 0.28	1.06 ± 0.32	<0.001
RC, mmol/l	0.64 (0.47, 0.83)	0.37 (0.29, 0.42)	0.55 (0.51, 0.59)	0.72 (0.67, 0.77)	1.01 (0.90, 1.20)	<0.001
Glu, mmol/l	7.72 ± 2.80	7.22 ± 2.62	7.49 ± 2.71	7.73 ± 2.76	8.41 ± 2.97	<0.001
UA, μmol/L	304 (254, 364)	290 (243, 347)	296 (248, 354)	309 (255, 370)	324 (270, 378)	<0.001
HbA1c, %	8.34 ± 2.24	8.02 ± 2.15	8.09 ± 2.11	8.44 ± 2.34	8.79 ± 2.27	<0.001
sCr, μmol/L	74.27 ± 13.93	75.36 ± 13.67	73.93 ± 14.21	73.84 ± 14.21	74.00 ± 13.54	0.135
TP, g/L	67.43 ± 5.85	67.12 ± 5.98	67.60 ± 5.92	67.07 ± 5.58	67.92 ± 5.89	0.106
Albumin, g/L	41.51 ± 3.74	41.41 ± 3.91	41.48 ± 3.63	41.28 ± 3.80	41.85 ± 3.59	0.121
ACR, mg/g	10.10 (5.20, 16.13)	10.30 (5.90, 16.60)	10.10 (4.80, 15.90)	9.90 (4.65, 15.60)	10.32 (5.66, 17.30)	0.751
eGFR, ml/min/1.73 m^2^	78.44 ± 8.95	76.70 ± 7.98	77.81 ± 8.46	78.83 ± 8.94	80.34 ± 9.88	<0.001
Hypertension, *n* (%)	1,024 (48.26)	280 (55.12)	276 (51.40)	245 (45.29)	223 (41.60)	<0.001
ACEI/ARB use, *n* (%)	373 (17.58)	108 (21.26)	97 (18.06)	96 (17.75)	72 (13.43)	0.001
Insulin therapy, *n* (%)	708 (33.37)	187 (36.81)	184 (34.26)	176 (32.53)	161 (30.04)	0.017
Fibrate/statin use	330 (15.55)	106 (20.87)	100 (18.62)	63 (11.65)	61 (11.38)	<0.001
SGLT2i/GLP-1RA use	214 (10.09)	46 (9.06)	64 (11.92)	49 (9.06)	55 (10.26)	0.919

SBP, systolic blood pressure; DBP, diastolic blood pressure; BMI, body mass index; TC, total cholesterol; TG, triglycerides; LDL-C, low-density lipoprotein cholesterol; HDL-C, high-density lipoprotein cholesterol; RC, remnant cholesterol; Glu, fasting blood-glucose; UA, uric acid; HbA1c, glycated hemoglobin A1c; sCr, serum creatinine; TP, total protein; ACR, albumin-to-creatinine ratio; eGFR, estimated glomerular filtration rate; ACEI, angiotensin-converting enzyme inhibitor; ARB, angiotensin II receptor blocker; SGLT2i, sodium-glucose cotransporter 2 inhibitor; GLP-1RA, glucagon-like peptide-1 receptor agonist.

### Association between RC levels and incident DKD risk

Multivariable Cox regression analysis was conducted to evaluate the association between RC levels and the risk of incident DKD (Table 2). When RC was analyzed as a continuous variable, each unit increase in RC was significantly associated with a higher risk of DKD in the age- and sex-adjusted model (Model 1: HR, 1.198; 95% CI, 1.037–1.385; *p* = 0.014). However, this association was attenuated and no longer statistically significant after further adjustment for clinical covariates in Model 2 and Model 3.

**Table 2:** HR for incident DKD associated with RC as a Continuous variable and by quartiles.

	Model 1			Model 2			Model 3		
RC level	HR	95%CI	*P* value	HR	95%CI	*P* value	HR	95%CI	*P* value
Per unit increase	1.198	1.037–1.385	0.014	1.062	0.910–1.241	0.445	1.073	0.921–1.249	0.364
Quartile 1	Ref			Ref			Ref		
Quartile 2	1.400	1.076–1.821	0.012	1.454	1.115–1.895	0.006	1.478	1.133–1.929	0.004
Quartile 3	1.457	1.108–1.915	0.007	1.429	1.084–1.883	0.011	1.479	1.118–1.958	0.006
Quartile 4	1.537	1.159–2.037	0.003	1.401	1.053–1.863	0.020	1.461	1.092–1.953	0.011
*P* for trend	0.011			0.022			0.012		

DKD, diabetic kidney disease; RC, remnant cholesterol; HR, hazard ratios; CI, confidence intervals; SBP, systolic blood pressure; BMI, body mass index; HbA1c, glycated hemoglobin A1c; ACR, albumin-to-creatinine ratio; sCr, serum creatinine; SGLT2i, sodium-glucose cotransporter 2 inhibitor; GLP-1RA, glucagon-like peptide-1 receptor agonist.

Model 1: adjusted for age and sex.

Model 2: model 1 + adjusted for SBP, BMI, ACR, sCr, HbA1c.

Model 3: model 2 + adjusted for diabetic duration, hypertension, Fibrate/statin use and SGLT2i/GLP-1RA use.

When RC was categorized into quartiles, higher RC levels were consistently associated with an increased risk of DKD across all models. In the fully adjusted model (Model 3), compared with participants in the lowest quartile (Q1), those in Q2, Q3, and Q4 had significantly higher risks of DKD (HR for Q2, 1.478; 95% CI, 1.133–1.929; *p* = 0.004; HR for Q3, 1.479; 95% CI, 1.148–1.958; *p* = 0.006; HR for Q4, 1.461; 95% CI, 1.092–1.953; *p* = 0.011). Notably, although a statistically significant linear trend was observed across RC quartiles in all the three models, the hazard ratios for Q2 to Q4 were numerically similar and did not exhibit a clear linear dose–response pattern.

**Figure 2:**
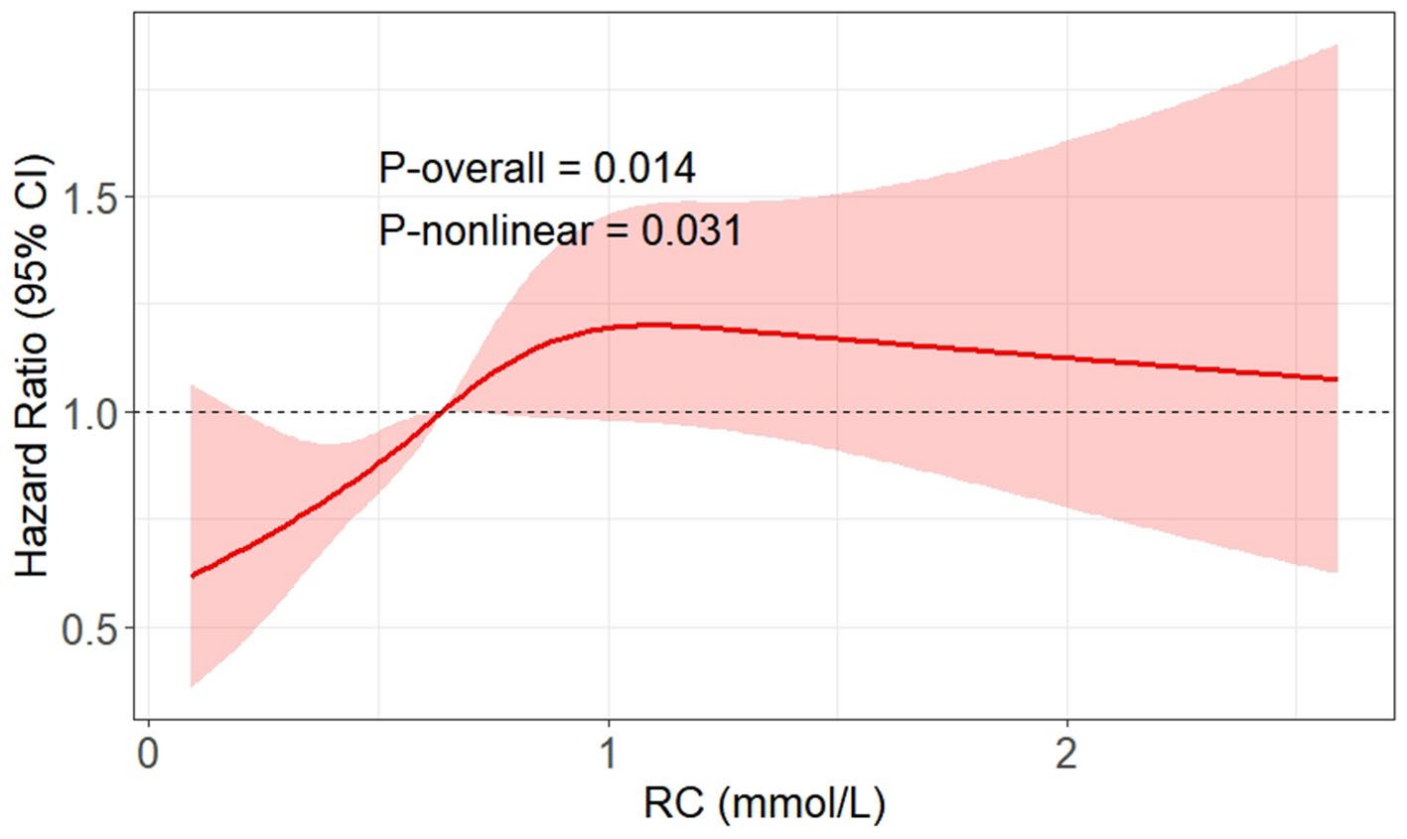
RCS plot showing the non-linear dose–response relationship between RC levels and risk of incident DKD adjusted for multiple confounders. The multiple confounders included age, sex, SBP, BMI, ACR, sCr, HbA1c, diabetic duration, hypertension, fibrate/statin use, and SGLT2i/GLP-1RA use. The reference value (HR = 1) was set at the median RC level (0.64 mmol/L). DKD, diabetic kidney disease; SBP, systolic blood pressure; BMI, body mass index; RC, remnant cholesterol; HbA1c, glycated hemoglobin A1c; sCr, serum creatinine; ACR, albumin-to-creatinine ratio; SGLT2i, sodium-glucose cotransporter 2 inhibitor; GLP-1RA, glucagon-like peptide-1 receptor agonist.

RCS analysis was performed to further assess the potential nonlinearity of this association (Figure 2). The spline curve demonstrated a nonlinear relationship between RC and DKD risk (P for nonlinearity = 0.031), characterized by an initial steep increase in risk followed by a flattening of the curve at higher RC concentrations. The overall association remained statistically significant (P overall = 0.014).

### Variable selection for incident DKD risk prediction using RSF algorithm

**Figure 3:**
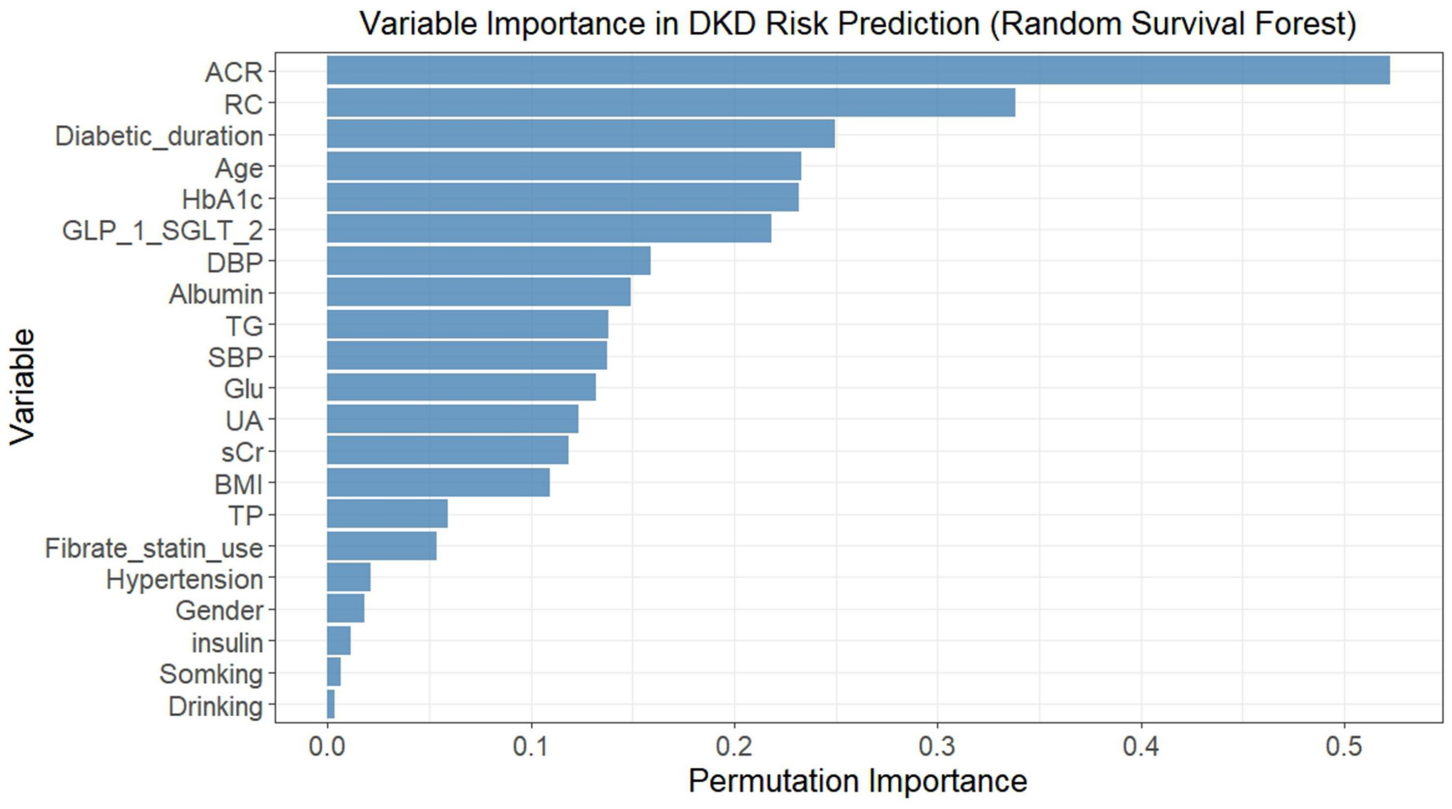
Permutation importance of variables in predicting incident DKD risk using random survival forest. Variables were ranked by permutation importance (higher values indicated greater importance in predicting DKD risk). The model included 21 baseline variables, and permutation importance was calculated by randomly permuting each variable’s values and measuring the decrease in model performance (concordance index). DKD, diabetic kidney disease; SBP, systolic blood pressure; DBP, diastolic blood pressure; BMI, body mass index; TG, triglycerides; RC, remnant cholesterol; Glu, fasting blood-glucose; UA, uric acid; HbA1c, glycated hemoglobin A1c; sCr, serum creatinine; TP, total protein; ACR, albumin-to-creatinine ratio; SGLT2i, sodium-glucose cotransporter 2 inhibitor; GLP-1RA, glucagon-like peptide-1 receptor agonist.

In light of the nonlinear relationship between RC and DKD risk indicated by the RCS analysis, and considering that the dataset involved time-to-event outcomes and multiple correlated clinical variables, a RSF algorithm was applied to explore potential predictors of incident DKD from 21 candidate variables. The permutation importance ranking was shown in Figure 3. Among all variables, ACR demonstrated the highest permutation importance, followed by RC and diabetic duration. Based on both permutation importance values and the clinical relevance reported in previous studies, variables with permutation importance > 0.1 were selected as predictors for DKD risk. A total of 14 variables met this criterion, including SBP, DBP, BMI, RC, TG, Glu, UA, HbA1c, sCr, ACR, age, diabetic duration, albumin, and SGLT2i/GLP-1RA use.

### Assessment of collinearity among selected variables

**Figure 4:**
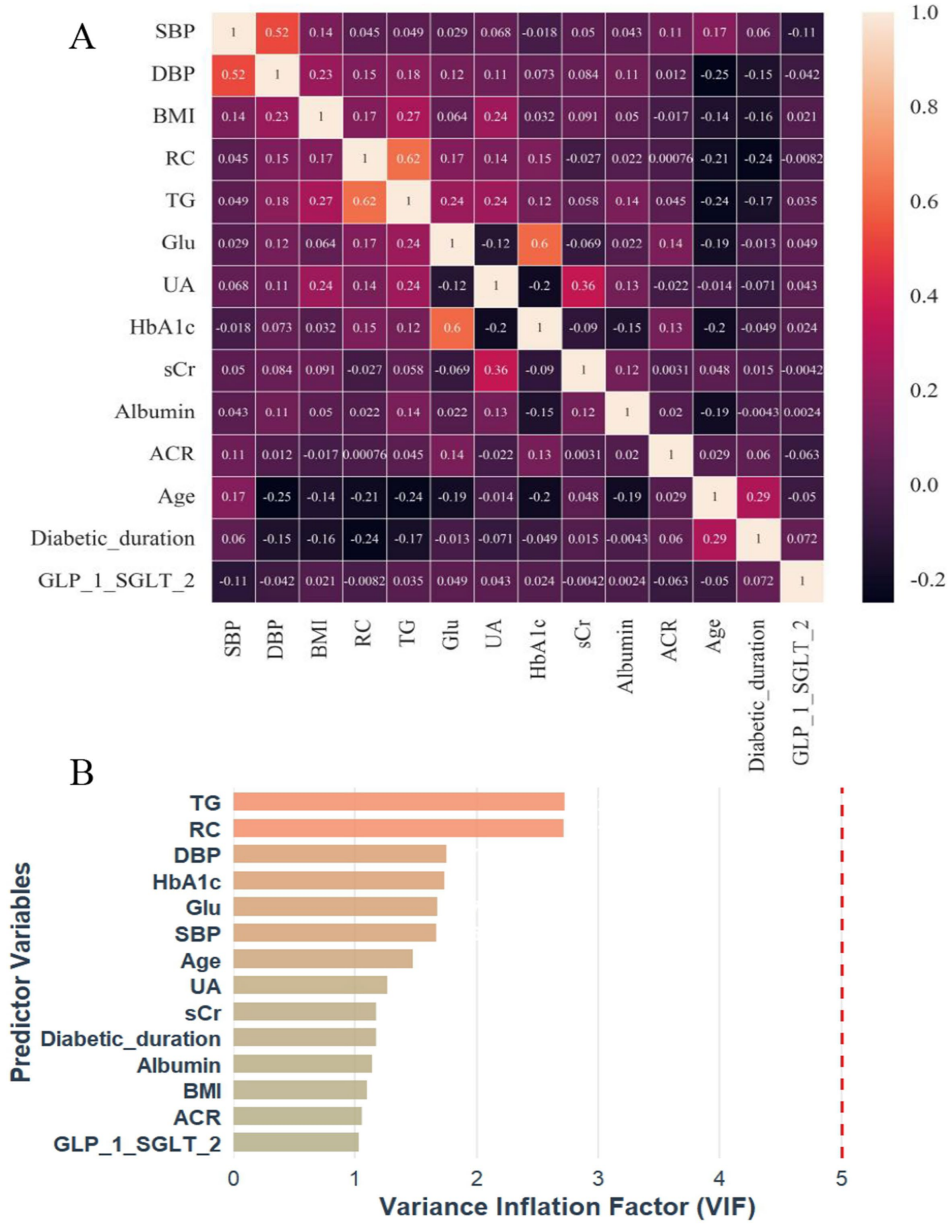
Correlation heatmap **(A)** and VIF plot **(B)** for multicollinearity assessment of 14 RSF-selected predictor variables in incident DKD risk prediction. DKD, diabetic kidney disease; SBP, systolic blood pressure; DBP, diastolic blood pressure; BMI, body mass index; TG, triglycerides; RC, remnant cholesterol; Glu, fasting blood-glucose; UA, uric acid; HbA1c, glycated hemoglobin A1c; sCr, serum creatinine; ACR, albumin-to-creatinine ratio; SGLT2i, sodium-glucose cotransporter 2 inhibitor; GLP-1RA, glucagon-like peptide-1 receptor agonist; VIF, variance inflation factor.

To evaluate potential collinearity among the 14 variables selected from the RSF analysis, spearman’s rank correlation coefficient was first used to examine their pairwise correlations (Figure 4A). Overall, no strong correlations were observed, and most variable pairs demonstrated either no significant association or only weak correlations. The strongest correlations were observed between TG and RC (*r* = 0.62, *p* < 0.001), followed by HbA1c and fasting glucose (*r* = 0.60, *p* < 0.001) and between SBP and DBP (*r* = 0.52, *p* < 0.001). Variance inflation factors (VIFs) were subsequently calculated to further assess multicollinearity (Figure 4B). All VIF values were below the commonly used threshold of 3, with the highest observed for TG (VIF = 2.74).

### Model development and performance evaluation

A predictive model for incident DKD was constructed using the RSF algorithm, which integrated the 14 pre-selected variables. Model performance was evaluated in terms of discrimination, calibration, and clinical utility.

**Figure 5:**
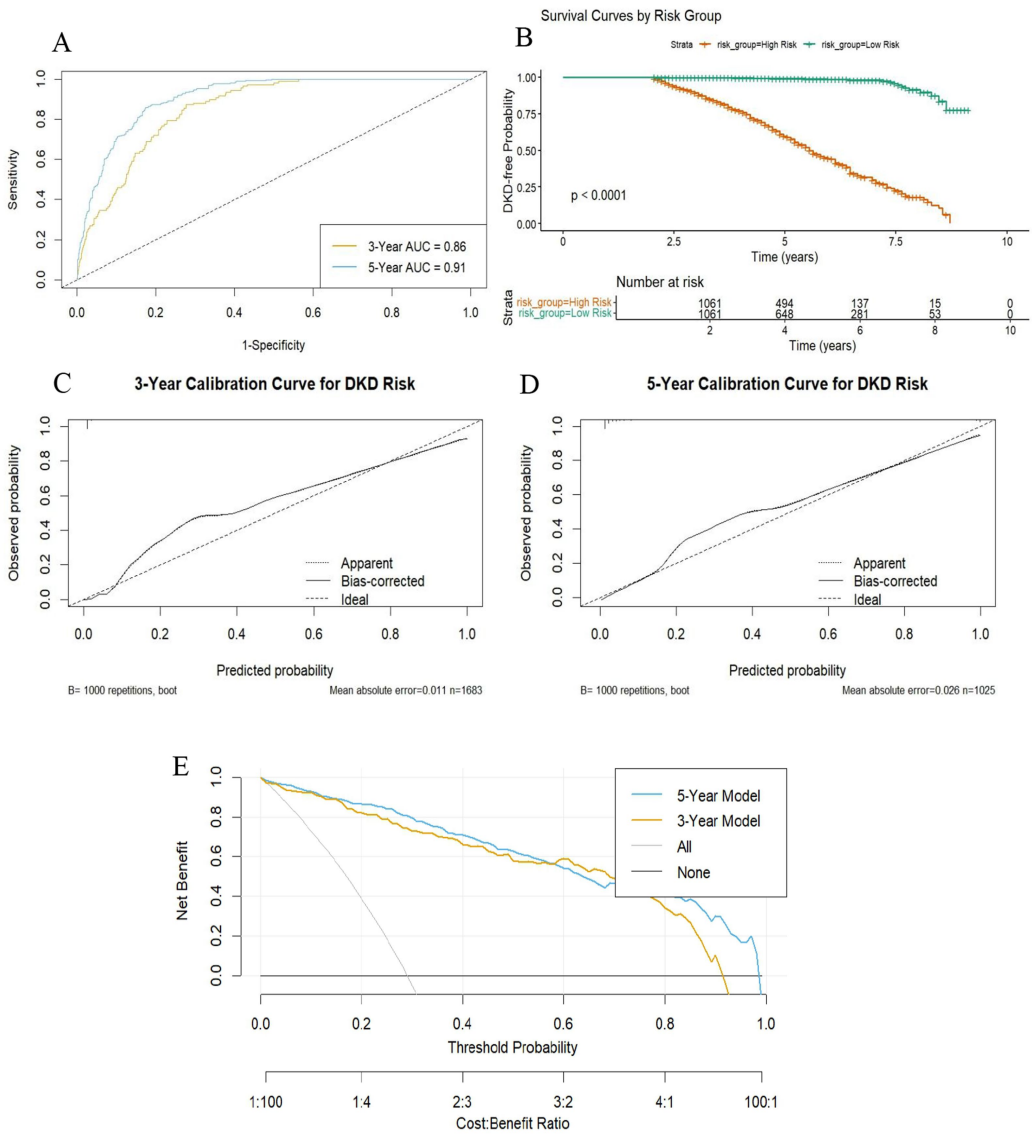
Performance evaluation of the DKD risk prediction model based on random survival forest. **(A)** Time-dependent ROC curves showing discriminatory power at 3 and 5 years; **(B)** Kaplan–Meier curves comparing cumulative DKD risk between high- (≥median model score) and low-risk (<median model score) groups; **(C,D)** Calibration curves evaluating agreement between predicted and observed risk at 3 and 5 years, validated by 1,000 bootstrap resamples. **(E)** DCA curves showing net clinical benefit of the model at 3 and 5 years, compared to “treat all” and “treat none” strategies. DKD, diabetic kidney disease; AUC, area under the curve; DCA, decision curve analysis.

Discrimination ability was assessed using time-dependent ROC curves and risk stratification. As shown in Figure 5A, the model achieved AUC values of 0.86 for 3-year and 0.91 for 5-year DKD prediction. Kaplan–Meier survival analysis further confirmed effective risk stratification, with clear separation between high- and low-risk groups defined by the median RSF score (Figure 5B) and a statistically significant difference in cumulative DKD incidence (log-rank *p* < 0.001). To delineate time-specific risk stratification by the RSF model, Kaplan–Meier curves were stratified at 3-year and 5-year horizons according to the median predicted risk (Supplementary Figures S1A,B). Participants in the high-risk group exhibited significantly elevated cumulative DKD incidence compared to the low-risk group at both time points (Log-rank *p* < 0.001).

Calibration accuracy was examined using calibration plots with 1,000 bootstrap repetitions. For 3-year prediction (Figure 5C), the model demonstrated close agreement between predicted and observed risks (mean absolute error = 0.011; mean squared error = 0.00087; 90th percentile absolute error = 0.019; *n* = 1,683). For 5-year prediction (Figure 5D), calibration remained acceptable, although prediction errors increased slightly (mean absolute error = 0.026; mean squared error = 0.0015; 90th percentile absolute error = 0.061; *n* = 1,025).

Clinical utility was evaluated by decision curve analysis (Figure 5E). Both the 3-year and 5-year models provided greater net benefit than the “treat-all” and “treat-none” strategies across a wide range of threshold probabilities. The 5-year model consistently demonstrated a marginally superior net benefit compared to the 3-year model.

## Discussion

This retrospective cohort study demonstrated that higher levels of RC were independently associated with an increased risk of incident DKD in patients with T2D, and that this relationship was nonlinear, with a steep rise in risk at lower RC levels followed by a plateau at higher concentrations. Furthermore, a RSF model incorporating RC and other routine clinical variables was constructed, and it achieved high discriminative performance for 3-year and 5-year DKD prediction, showed good calibration, and provided net clinical benefit across a range of decision thresholds. These findings might have a potential utility for enhancing individualized DKD risk stratification in patients with T2D.

RC, reflecting cholesterol content in triglyceride-rich remnant lipoproteins, has garnered increasing attention as an independent predictor of atherosclerotic and microvascular outcomes (29–31). A large Korean cohort of type 2 diabetes patients (*n* = 1.96 million participants) demonstrated that individuals in the highest RC quartile exhibited significantly elevated risks for myocardial infarction (HR = 1.28, 95%CI:1.25–1.31) and ischemic stroke (HR = 1.22, 95%CI:1.20–1.25), independent of LDL-C and statin use (29). Similarly, large cohort studies in European populations confirmed remnant cholesterol as a strong independent risk factor for peripheral artery disease, myocardial infarction, and ischemic stroke (30). Notably, its association with peripheral artery disease was substantially stronger than with myocardial infarction or stroke, and it consistently demonstrated greater predictive value than traditional lipid parameters in multivariable-adjusted models (30). Mechanistically, RC particles promote vascular injury by activating vascular endothelia and triggering systemic inflammation (e.g., elevating hsCRP) (32–34). The EPIC-Norfolk study confirmed this inflammatory effect is unique to RC (not observed with LDL-C), mediated through small VLDL particles and apoC-III (32). RC’s synergy with hyperglycemia-induced endothelial dysfunction highlights its pathological relevance in microvascular disease, providing a mechanistic rationale for RC-related complications extending beyond macrovascular pathology (32). These findings establish a pathophysiological foundation for exploring RC’s role in DKD, where microvascular injury is central to pathogenesis.

To evaluate the association between RC and incident DKD risk, this study employed RC quartile-based stratification, revealing distinctive baseline patterns. Interestingly, an inverse relationship between age and RC quartiles was observed, with participants in higher RC categories tending to be younger. This pattern was consistent with epidemiological data showing that RC levels often peak in younger to middle-aged adults before declining in older populations, potentially owing to age-related changes in lipoprotein metabolism or treatment patterns (35, 36). Moreover, the lower prevalence of hypertension among individuals with higher RC levels may be partially explained by their younger age, as advanced age is a strong independent risk factor for hypertension (37, 38). Alternatively, it is possible that differences in statin use, lifestyle factors, or the presence of comorbid conditions such as kidney impairment in older individuals contribute to these patterns. These observations underscore the importance of considering age and comorbid profiles when interpreting RC-associated risk, and suggest that future studies should explore age-stratified associations and the influence of therapeutic interventions on remnant cholesterol dynamics.

In the present study, higher RC levels were significantly associated with increased incident DKD risk (P for trend < 0.05 across all adjusted models), which was broadly consistent with prior evidence linking elevated RC to adverse renal outcomes (39, 40). Of note, the loss of significance for RC as a continuous variable in Models 2 and 3 likely reflected the nonlinear relationship between RC and DKD risk, as evidenced by the RCS analysis. The plateau effect at higher RC concentrations indicated that per-unit increments in RC beyond the threshold (Q1) did not proportionally increase risk. Conversely, categorical analysis captured the threshold effect: participants exceeding the lowest quartile (Q1) exhibited a consistently elevated risk (HR ≈ 1.5), regardless of further RC elevation. This explained the persistent significance in quartile-based models. Additionally, Population-based analyses have shown that higher RC is associated with lower eGFR and a higher prevalence of albuminuria after adjustment for conventional lipids and cardiometabolic risk factors (39). In longitudinal settings, RC has also been related to faster renal disease progression and a higher risk of end-stage kidney disease among patients with diabetic nephropathy (40). Although most previous investigations examined chronic kidney disease of mixed etiologies, the present findings extended these observations to incident DKD in a Chinese T2D cohort. This is consistent with the well-established role of RC as a residual cardiovascular risk factor (14–17) and supports the concept that RC may also contribute to microvascular complications in diabetes.

Several mechanisms may underlie the observed link between RC and DKD. RC, as a measure of cholesterol content in triglyceride-rich remnant lipoproteins, reflects an atherogenic lipid burden not captured by LDL-C (29–31). Triglyceride-rich remnant particles can penetrate the glomerular filtration barrier and be taken up by mesangial and proximal tubular cells, leading to intrarenal lipid accumulation and “lipotoxicity,” which provoke mitochondrial dysfunction, endoplasmic reticulum stress, and excessive reactive oxygen species generation (12, 13, 41, 42). These metabolic insults activate inflammatory and fibrotic pathways, including NF-κB and TGF-β signaling, resulting in mesangial expansion, glomerulosclerosis, and tubulointerstitial fibrosis. RC-induced lipotoxic stress also amplifies systemic and local low-grade inflammation—particularly via small VLDL and apoC-III–mediated pathways—thereby aggravating endothelial dysfunction and microvascular injury that are central to DKD pathogenesis (32, 43). Furthermore, RC-related metabolic disturbances may synergize with hyperglycemia and insulin resistance to enhance oxidative and inflammatory stress within glomerular cells, accelerating renal damage progression (10–12).

In this study, a steep-rise–plateau pattern between RC and DKD risk was observed in the RCS analysis, it might reflect a biological threshold beyond which additional remnant lipoprotein accumulation exerts diminishing renal toxicity. At lower to moderate RC levels, increasing concentrations of triglyceride-rich remnant particles can markedly amplify oxidative stress, endothelial dysfunction, and lipid deposition within glomerular and tubular compartments, triggering inflammatory and fibrotic cascades that accelerate renal injury (10, 11, 13, 41, 42). However, once these pathogenic pathways are maximally activated, further increases in RC may confer limited additional risk, leading to a saturation-like plateau. Similar threshold phenomena have been reported for other metabolic risk markers such as the triglyceride–glucose index in DKD (28), suggesting that early-stage lipid accumulation and endothelial activation are key rate-limiting steps in the transition from metabolic disturbance to overt renal damage. This nonlinear pattern may underscore the potential benefit of early RC control before reaching the inflection point where irreversible microvascular injury ensues.

However, some studies have reported attenuated or non-significant associations between RC and renal outcomes (39, 44), emphasizing potential effect modification by study design, endpoint definition, and population characteristics. In an NHANES-based cross-sectional analysis (39), higher remnant cholesterol was associated with lower eGFR in dose–response fashion, but the link with albuminuria lost statistical significance after full adjustment (OR 1.24; 95% CI: 0.95–1.61). Similarly, prognostic analysis of the ACCORD cohort revealed that RC had no statistically significant association with progression to renal failure (*p* = 0.621) (38). These discrepancies may reflect differences in outcome definitions, duration of follow-up, ethnic and metabolic backgrounds, as well as varying degrees of concomitant lipid-lowering therapy. Taken together, these findings suggest that the prognostic value of RC may vary by renal endpoint and population context, underlining the need for prospective, standardized research across diverse cohorts.

This study has several limitations. First, its retrospective, single-center design may limit causal inference and restrict the generalizability of the findings to broader T2D populations, as patient characteristics, clinical practices, and local healthcare patterns may differ from those in other regions or settings. Second, on one hand, residual confounding cannot be entirely excluded, and certain potentially relevant variables—such as dietary habits, inflammatory biomarkers, socioeconomic factors, and genetic predispositions—were not available in the dataset; on the other hand, despite multivariable adjustment, residual confounding persists due to imbalances in antihypertensive/lipid-lowering drug usage across RC quartiles. Third, RC was calculated indirectly from TC, LDL-C, and HDL-C rather than directly measured, which is a common and validated approach in epidemiological research but does not capture remnant lipoprotein particle number, size, or composition, potentially limiting mechanistic interpretation. Finally, the prediction model underwent internal validation but lacked external validation in independent cohorts. For a time-dependent model, dividing the dataset into separate training and validation sets would have yielded a relatively small number of DKD cases in each subset, as only events occurring within the time window are eligible for analysis. This scarcity of events per analytical window could compromise parameter estimation stability. Therefore, model construction was performed using the entire dataset, and calibration was assessed using bootstrap resampling with 1,000 iterations in this study. External validation in larger, multi-center, independent populations is warranted to confirm robustness and applicability.

## Conclusion

In conclusion, this study found that RC was independently associated with incident DKD risk in T2D, with a nonlinear pattern characterized by a steep increase in risk at moderate RC levels, followed by a plateau at higher concentrations. In addition, an RSF-based prediction model integrating RC with other routinely available clinical variables showed good discrimination, acceptable calibration, and potential clinical utility. These findings indicate that RC may be a useful variable for more refined DKD risk stratification in T2D, and that machine learning approaches could provide a feasible strategy for developing clinically applicable prediction tools.

## Data Availability

The raw data supporting the conclusions of this article will be made available by the authors, without undue reservation.

## Glossary

ACEI: Angiotensin converting enzyme inhibitor
ARB: Angiotensin receptor blocker
ACR: Albumin-to-creatinine ratio
BMI: Body mass index
DKD: Diabetes kidney disease
DBP: Diastolic blood pressure
eGFR: Estimated glomerular filtration rate
RC: Remnant cholesterol
GLP-1RA: Glucagon-like peptide-1 receptor agonists
HbA1c: Glycated hemoglobin
HDL-C: High-density lipoprotein cholesterol
HR: Hazard ratios
LDL-C: Low-density lipoprotein cholesterol
MSS: Maximum selected statistics
RCS: Restricted cubic spline
SBP: Systolic blood pressure
SGLT2i: Sodium-glucose cotransporter-2 inhibitors
T2D: Type 2 diabetes
RSF: Random survival forest
ROC: Receiver operating characteristic
VIF: Variance inflation factors
DCA: Decision curve analysis
VLDL: Very-low-density lipoprotein cholesterol
IDL-C: Intermediate-density lipoprotein cholesterol
