# Supplementary material for: Remnant cholesterol for diabetic kidney disease risk stratification in type 2 diabetes: a machine learning-based prevention tool
Source: Front Nutr. 2025 Nov 11;12:1697943. doi: 10.3389/fnut.2025.1697943 (PMC12643847; doi:10.3389/fnut.2025.1697943)
Supplement: Supplementary file 1 [file Supplementary_file_1.docx]

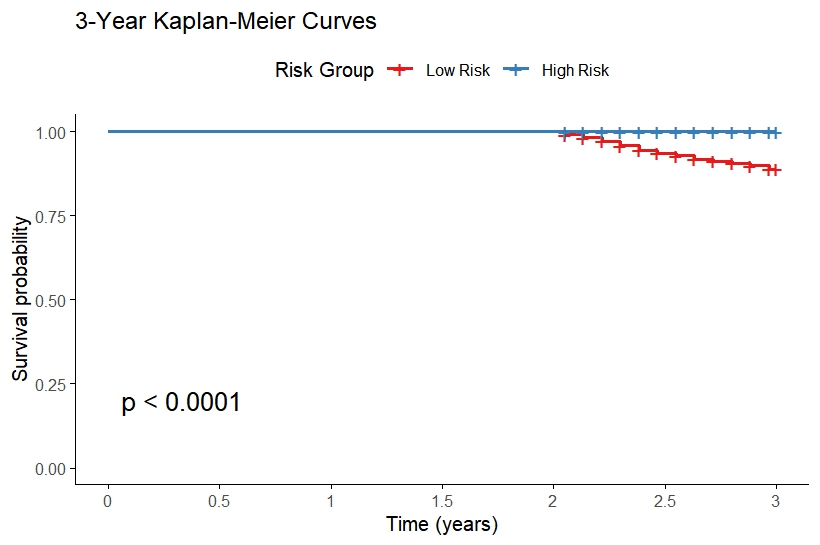

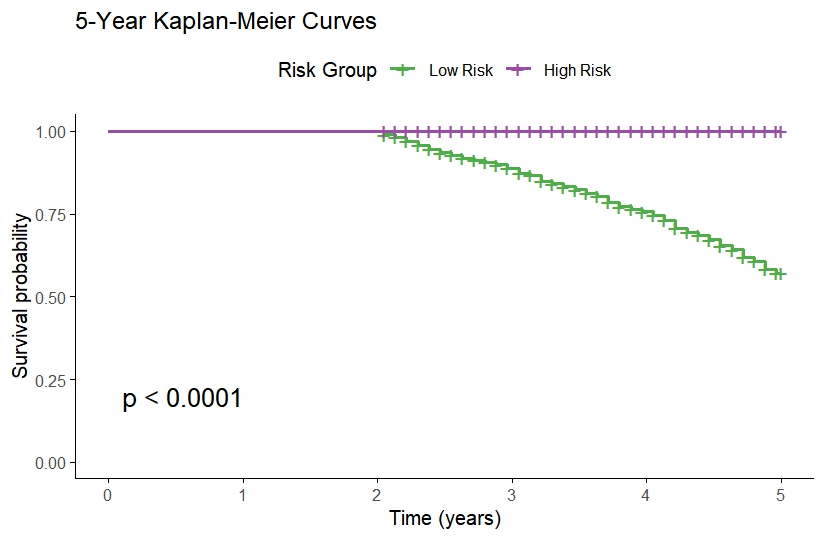


A

B

Supplementary Figure 1. Time-stratified Kaplan-Meier curves for DKD incidence. (A) 3-year cumulative incidence stratified by median predicted risk from the RSF model. (B) 5-year cumulative incidence stratified by median predicted risk from the RSF model. DKD, diabetic kidney disease; RSF, random survival forest.
